# Differences in the Genital Microbiota in Women Who Naturally Clear *Chlamydia trachomatis* Infection Compared to Women Who Do Not Clear; A Pilot Study

**DOI:** 10.3389/fcimb.2021.615770

**Published:** 2021-04-12

**Authors:** Patricia Dehon Mott, Christopher M. Taylor, Rebecca A. Lillis, Caleb M. Ardizzone, Hannah L. Albritton, Meng Luo, Kaitlyn G. Calabresi, David H. Martin, Leann Myers, Alison J. Quayle

**Affiliations:** ^1^ Department of Microbiology, Immunology and Parasitology, Louisiana State University Health Sciences Center, New Orleans, LA, United States; ^2^ Division of Infectious Diseases, Department of Medicine, Louisiana State University Health Sciences Center, New Orleans, LA, United States; ^3^ Department of Biostatistics and Data Science, Tulane University School of Public Health and Tropical Medicine, Tulane University, New Orleans, LA, United States

**Keywords:** *Chlamydia trachomatis*, bacterial vaginosis, interferon-γ, proinflammatory cytokines, microbiome, natural clearance, women

## Abstract

*In vitro* studies indicate IFNγ is central to *Chlamydia trachomatis* (Ct) eradication, but its function may be compromised by anaerobes typically associated with bacterial vaginosis (BV), a frequent co-morbidity in women with Ct. Here we investigated the associations between natural clearance of cervical Ct infection, the vaginal microbiome, and the requirements for IFNγ by evaluating the vaginal microbial and cytokine composition of Ct treatment visit samples from women who cleared Ct infection in the interim between their Ct screening and Ct treatment visit. The pilot cohort was young, predominantly African American, and characterized by a high rate of BV that was treated with metronidazole at the Ct screening visit. The rate of natural Ct clearance was 23.6% by the Ct treatment visit (median 9 days). 16S rRNA gene sequencing revealed that metronidazole-treated women who had a *Lactobacillus* spp.-dominant vaginal microbiota (CST 2 or 3) at the Ct treatment visit, were more prevalent in the Ct clearing population than the non-clearing population (86% v. 50%). *L. iners* (CST2) was the major *Lactobacillus* spp. present in Ct clearers, and 33% still remained anaerobe-dominant (CST1). Vaginal IFNγ levels were not significantly different in Ct clearers and non-clearers and were several logs lower than that required for killing Ct *in vitro.* An expanded panel of IFNγ-induced and proinflammatory cytokines and chemokines also did not reveal differences between Ct clearers and non-clearers, but, rather, suggested signatures better associated with specific CSTs. Taken together, these findings suggest that BV-associated bacteria may impede Ct clearance, but a *Lactobacillus* spp.-dominant microbiome is not an absolute requirement to clear. Further, IFNγ may be required at lower concentrations than *in vitro* modeling indicates, suggesting it may act together with other factors *in vivo*. Data also revealed that the vaginal bacteria-driven inflammation add complexity to the genital cytokine milieu, but changes in this microbiota may contribute to, or provide cytokine biomarkers, for a shift to Ct clearance.

## Introduction

*Chlamydia trachomatis* (Ct) infection is the most prevalent national notifiable infectious disease in the US, and the most common sexually transmitted bacterial infection worldwide (Newman et al., 2015). In some women the bacteria will ascend from the cervix, the primary site of infection, into the uterus and fallopian tubes where chronic infection can lead to severe reproductive pathology (Brunham and Rey-Ladino, 2005; Brunham and Rekart, 2009). While public health initiatives have increased Ct screening and treatment rates, leading to decreased upper tract pathologies, efforts to control the infection are mitigated by a lack of global access to screening, the asymptomatic nature of the infection and high reinfection rate (Workowski et al., 2010; Datta et al., 2014). Vaccine initiatives are being embraced, but there has been a historic absence of a readily identifiable cohort to help inform correlates of protection. A series of studies from one US sexually transmitted infection (STI) Clinic, however, revealed first that ~20% of women screened for Ct naturally cleared the infection in the short interim between screening and antibiotic treatment visits and second, that these women were also significantly protected from reinfection (Geisler et al., 2008; Geisler et al., 2013). Thus, these studies revealed a key patient group that may help identify the genital immune and environmental correlates of resolution and subsequent protection from reinfection. The data also reveal that the rate of natural clearance may be bi-phasic since a large long-term natural history study previously showed that only 50% of women will naturally resolve Ct infection in one year and 80% in two years (Morre et al., 2002; Molano et al., 2005). This dichotomy needs further investigation.

*In vitro* and animal studies have reiterated a central role for the cytokine interferon gamma (IFNγ) in the resolution of Ct, an obligate intracellular pathogen (Carlin et al., 1989; Rank et al., 1992; Beatty et al., 1994a; Gondek et al., 2009). Modeling in human endocervical epithelial cells, the primary site of Ct infection, indicates IFNγ induces the tryptophan-degrading enzyme indoleamine 2,3-dioxygenase (IDO-1) (Beatty et al., 1994a; Xie et al., 2002). Since Ct cannot synthesize tryptophan *de novo*, the bacteria can be eradicated by starvation. However, the vast majority of human genital Ct isolates express a tightly regulated tryptophan synthase (*trpBA*) that can salvage indole to make tryptophan (Caldwell et al., 2003). This would suggest indole salvage has been selected by genital serovars of Ct as one mechanism to evade eradication. Since neither Ct nor its human host make indole, Caldwell et al. hypothesized it could be provided by bacteria comprising the bacterial vaginosis (BV) microbiome or community state type (CST), typified by an abundance of anaerobes which includes indole producers, and low levels of *Lactobacilli.* A clinical BV diagnosis is generally made using Amsel clinical criteria (BV-Amsel) (Amsel et al., 1983), or by the Nugent scoring system (BV-Nugent) (Nugent et al., 1991) which captures bacterial morphotypes on a Gram stain; vaginal microbiota can also be molecularly classified by 16S rRNA gene sequence characterization of vaginal bacterial CSTs (BV-CST) (Mckinnon et al., 2019). Using CST analyses, vaginal bacterial communities are more specifically classified based on species dominance and relative abundance (Redelinghuys et al., 2020), and ‘optimal’ and ‘non-optimal communities’ defined by these molecular techniques broadly overlap with BV defined by classical methods, although are still considered distinct (Mckinnon et al., 2019).

BV is the most frequent cause of vaginal discharge and malodor (Allsworth and Peipert, 2007) and is found in 20-65% of Ct-infected women (Wiesenfeld et al., 2003; Ficarra et al., 2008; Balle et al., 2018; Filardo et al., 2019). A BV diagnosis by Amsel criteria, used as a point-of-care test in many STI clinics including our own, prompts physicians to prescribe oral metronidazole, metronidazole gel or clindamycin cream at a Ct screening visit. Metronidazole treatment is effective in most, although not all women, with up to 84% clinical cure rates at 1 month (Oduyebo et al., 2009). However, BV commonly reoccurs and requires retreatment; 58% of treated women will have a recurrence of BV-Amsel by 12 months (Bradshaw et al., 2006). Recent, extensive studies in high-risk young women have shown that BV can drive a local cervicovaginal pro-inflammatory milieu (Sturm-Ramirez et al., 2000; Anahtar et al., 2015; Jespers et al., 2017; Redelinghuys et al., 2020), potentially confounding studies designed to investigate correlates of immunity to Ct. Thus, there is a need to better understand the interaction of the vaginal microbiome and Ct infection in women in order to optimize treatment and prevention of both the latter and BV.

The purpose of this pilot study was to determine the potential associations between the natural clearance of cervical Ct infection, the vaginal microbiome and requirements for IFNγ. We approached this by evaluating the vaginal microbial and cervicovaginal cytokine milieu of a cohort of Ct positive women who were returning to our New Orleans STI Clinic for Ct treatment approximately 9 days after a Ct screen. Since the Ct screening visit was concomitant with a BV diagnosis and immediate BV treatment, this allowed us to also determine whether BV treatment may play a role in natural clearance of Ct.

## Materials and Methods

### Study Population

Women aged 18-35 years and with a recent (≤1 month) positive cervical or urine-based Ct Hologic^®^ APTIMA^®^ nucleic acid amplification test (NAAT) were recruited into the study when they returned to LSU CrescentCare Sexual Health Center, New Orleans clinic to receive azithromycin treatment and counseling for their Ct infection. Study exclusion criteria were: a positive *Neisseria gonorrhea* NAAT at the Ct screen; pregnancy or miscarriage in last 2 months; self-reported sexual intercourse within the last 12 hours; current menstrual bleeding; antibiotic use in the last 8 weeks, with the exception of metronidazole treatment at the Ct screen; or documented infection with HIV. Participants also provided information on demographics, menstrual cycle and contraception, history of previous sexually transmitted infections and sexual behavior. Approval for the study was obtained from the LSUHSC Human Research Ethics Committee, and written informed consent was obtained from each patient. Fifty-seven women were recruited over 2 years and Ct treatment visit samples from 55 women were included in the demographic analysis of natural clearance; two patients were excluded due to initially overlooked exclusion criteria (miscarriage, recent antibiotic treatment).

### Collection and Processing of Clinical Specimens

Genital secretions were collected at the Ct treatment visit using protocols similar to those we previously described (Albritton et al., 2017). In brief, endocervical sampling included a (i) cervical cytobrush (endocervical microbiome); (ii) Dacron swab immersed in endocervical transport medium (*C. trachomatis* culture); and (iii) endocervical swab for Ct and *N. gonorrhoeae* NAAT testing. Vaginal sampling included a (i) Copan Swab placed in 5 ml AssayAssure (microbiome, *M. genitalium* PCR*);* (ii) cotton swab for pH and wet mount (Amsel and *T. vaginalis*); (iii) cotton swab used for slide preparation (Gram stain for Nugent scoring); and (iv) 3 ml sterile saline cervicovaginal lavage (CVL) (vaginal cytokines) which was immediately supplemented with a protease inhibitor tablet (Roche). All samples were immediately placed on ice after collection and processed within 2 hours, after which they were stored in appropriate aliquots at -80°C until analysis.

### STI Testing

*Chlamydia trachomatis* and *Neisseria gonorrhoeae* (Ng) were detected by the APTIMA Combo 2 test, a target amplification nucleic acid probe test that utilizes target capture for detection of Ct and Ng rRNA (Hologic). Viable Ct was determined by semi-quantitative culture for inclusion forming units (IFU) (Ficarra et al., 2008). BV was diagnosed in the clinic by the four Amsel criteria (discharge, pH, Whiff test and presence of Clue cells, ≥3=positive) and subsequently by morphology by a Gram stain and Nugent scoring (0-3=negative, 4-6=intermediate, 7-10=positive). *Trichomonas vaginalis* was detected by a wet mount in clinic and confirmed with the APTIMA NAAT test. DNA was extracted from vaginal swabs and used to detect *M. genitalium* using a real-time quantitative PCR (qPCR) targeting a 92-bp region of the MG190 gene as previously described (Dehon and Mcgowin, 2014). Vaginal and endocervical derived DNA were also used for microbiome analysis; 54 vaginal and 20 endocervical samples were available for microbiome analysis.

### Vaginal Cytokines

CVLs were centrifuged at 12,000 x g for 30 minutes at 4°C and supernatants filtered through a 0.45 μm syringe filter). IFNγ, IL-1α, IL-1β, IL-6, IL-17, IP-10, MIP-1α, MIP-1β, RANTES and TNFα were quantified by a cytometric bead array assay (MILLIPLEX MAP Immunology Multiplex Assay; Millipore, Billerica, MA) per manufacturer’s instructions and as previously described (Buckner et al., 2011; Buckner et al., 2013). Samples from 34 women were suitable for cytokine analysis. Cytokine measurements below the limit of detection were assigned to a value of half of the minimum detectable concentration for that cytokine. If >50% women had an undetectable response, this cytokine was excluded from analyses (MIP-1α, MIP-1β). Raw values were log_10_ transformed before statistical analysis.

### Vaginal and Cervical Microbiomes

DNA amplification was performed to prepare the sequencing library using the AccuPrime Taq high-fidelity DNA polymerase system (Thermo-Fisher/Invitrogen/Life Technologies) as previously described (Kozich et al., 2013). The 16S rDNA hypervariable region V4 was amplified using 20 ng of genomic DNA and gene-specific primers with the following Illumina adaptors: 5′-TCGTCGGCAGCGTCAGATGTGTATAAGAGACAGGTGCCAGCMGCCGCGGTAA-3′ (forward) and 5′-GTCTCGT GGGCTCGGAGATGTGTATAAGAGACAGGGACTACHVGGGTWTCTAAT-3′ (reverse). Purified amplicon DNA from the last step with 25 cycles of PCR was then amplified for 8 cycles using the following primers with different molecular indexes: 5′-AATGATACG GCGA CCACCGAGATCTACAC [i5] TCGTCGGCAGCGTC- 3′ (forward) and 5′- CAAGCAGAAGACGGCATACGAGAT [i7] GTCTCGTGGGCTCGG-3′ (reverse). Normalized and pooled libraries were then run using 2x250 bp paired-end sequencing on an Illumina MiSeq (Illumina) with a 500 cycle V2 full sequencing kit.

Raw sequences were processed using DADA2 (v1.16.0) (Callahan et al., 2016). Region specific primers were trimmed off and reads were truncated to 240 bp to remove low quality ends of reads. Error rates were learned and used by the dada algorithm to infer sequence variants over a subset of >1e+08 bases. Sequence variants were merged and merged amplicons outside of the expected 250-254 bp length were discarded. Chimeras were removed using the consensus method. Over 95.7% of sequences remained after chimera removal and were placed into a sequence table comprising 1137 sequence variants. Taxonomic assignment was performed using the SILVA v138 database for assignment down to species level when available (Quast et al., 2013). Four negative sequencing controls were used with the decontam prevalence method (Davis et al., 2018) which identified 26 of the 1137 sequence variants as contaminants that were removed, leaving 1111 sequence variants. The decontam prevalence method is known to miss potential contaminants that are present in the majority of real samples as well as negative controls (e.g. lab contaminants) so as an additional contaminant removal step, we removed any additional sequence variants that remained at over 5% relative abundance in the negative controls. This step removed 5 more sequence variants (one Genus *Akkermansia*, two Genus *Cetobacterium*, and two that were not classified at the Genus level) leaving 1106 sequence variants. Secondary data analysis was performed using Phyloseq (v1.32.0) (Mcmurdie and Holmes, 2013). Sequence variants that appeared in only one sample were removed by a prevalence filter leaving 250 sequence variants. Though the majority of sequence variants were removed by this filter, the total read count only dropped from 854,785 to 839,671. Hence, this filter removed only 1.77% of sequencing reads. An abundance filter was then used to remove sequence variants that comprised less than 1% of sequencing reads per sample resulting in 145 remaining sequence variants. The total number of sequencing reads remaining after this filter was 835,940, hence this filter removed only 0.44% of remaining reads. *Shuttleworthia* genus was renamed as BVAB1 and *Fastidiosipila* genus was renamed as *Mageeibacillus indolicus* in order to reflect more accurate taxonomic classifications recently reported in the vaginal microbiome literature (Austin et al., 2015; Wessels et al., 2017; Holm et al., 2020). These ASVs when searched using BLAST against the nr/nt database produced 100% identity matches to the newly named taxa. The taxonomic classifications were then agglomerated to Species level, thereby combining any sequence variants that were classified to the same Species. This resulted in the 144 sequence variants collapsing to 81 taxonomic classifications. Lastly, taxonomic classifications for which the Genus level was not identified by Silva were filtered out of the data leaving us with 74 taxonomic classifications. The sequence table was subsetted to just the 54 vaginal site samples and among them the read counts ranged from a minimum of 5255 to a maximum of 30272. The median read count was 11558.5 with an IQR of 6722.75.

### Clustering Into Community State Types

The Bray-Curtis distance was used to calculate pairwise sample distances between the 54 vaginal site samples. This distance matrix was denoised by selecting the most significant Principal Component Analysis (PCA) eigenvectors as described in (Digiulio et al., 2015). We used the Partitioning around mediods (pamPCoA) algorithm and based on the gap statistic, we determined the number of clusters to use (k = 3). Assigned clusters were plotted with Phyloseq and showed that CST3 was primarily dominated by *L. crispatus*, CST2 was primarily dominated by *L. iners*, and CST1 consisted of a broad spectrum of organisms consistent with BV.

### Cytokine Principal Component Analysis

A PCA was performed over the log_10_ transformed cytokine values using prcomp. The PCA Biplot colored by Ct Status and CST was produced using the fviz_pca_biplot function from factoextra v1.0.7 R package.

### Statistical Analyses

The Mann-Whitney test was used for continuous variables in **Table 1**. The Student’s t-test was used for cytokine comparisons in **Supplemental Tables 1** and **2**, Fisher’s exact test was used for differences in prevalence and treatment success (**Table 1**). Analyses were performed using Prism (version 8.4.2; GraphPad Software, Inc., La Jolla, CA).

**Table 1:** Demographic, clinical and behavioral characteristics by *Chlamydia trachomatis* clearance status.

Characteristic	Total (n = 55)	Cleared Ct (n = 13)	Persisting (n = 42)	p-value^a^
Age, median (range)	24 (18-35)	26 (18-30)	24 (18-35)	0.32^b^
Black Race, No (%)	42 (76.4)	9 (69.2)	33 (78.6)	0.48
≤ 1 partner in 30 days, No. (%)	43 (78.2)	11 (84.6)	32 (76.2)	0.71
Hormonal Contraception^c^, No. (%)	22 (40.0)	8 (61.5)	14 (33.3)	0.11
Mucopurulent cervicitis, No. (%)	16 (29.1)	2 (15.4)	14 (33.3)	0.30
Prior Ct, No. (%)	28 (50.9)	5 (38.5)	23 (54.8)	0.36
Coinfection, No. (%)				
None^d^	23 (41.8)	7 (53.8)	16 (38.1)	0.52
BV Amsel (3-4)	16 (29.1)	2 (15.4)	14 (33.3)	0.30
BV Nugent (7-10)	21 (38.2)	3 (23.1)	18 (42.3)	0.33
*T. vaginalis*	1 (1.8)	0 (0.0)	1 (2.4)	>0.99
Yeast	4 (7.3)	2 (15.4)	2 (4.8)	0.23
*M. genitalium*	7 (12.7)	0 (0.0)	7 (16.7)	0.18
HSV	2 (3.6)	1 (7.7)	1 (2.4)	0.42
Days to enrollment, median (range)	9 (4-31)	9 (7-23)	9 (4-31)	0.92^b^
BV Treated at Screen, No (%)	28 (50.9)	7 (53.8)	21 (50.0)^e^	>0.99
Metronidazole Rx/Adhered at Screen, No (%)	26 (47.3)	7 (53.9)	19 (45.2)^f^	0.76
Treatment success (Amsel 0-2)	22 (84.6)	6 (85.7)	16 (84.2)	>0.99
Treatment success (Nugent<7)	17 (65.4)	6 (85.7)	11 (57.9)	0.36
Treatment success (by CST) ^e^	15 (57.7)	6 (85.7)	9 (50%)	0.18

a Fisher’s exact test, unless otherwise indicated.

b Mann-Whitney test.

c Hormonal contraception includes Mirena, Depo-Provera, oral contraceptives, and Paragard.

d Coinfection includes BV-Amsel, BV-Nugent, Trichomonas vaginalis, yeast, Mycoplasma genitalium, HSV.

e Two patients in persisting group did not adhere to treatment.

f CST available on 25/26 of those treated for BV symptoms at screen.

## Results

### Over one in Five Women in a New Orleans STI Clinic Cohort Naturally Clear *C. trachomatis* Between Their Ct Screening and Ct Treatment

**Figure 1:**
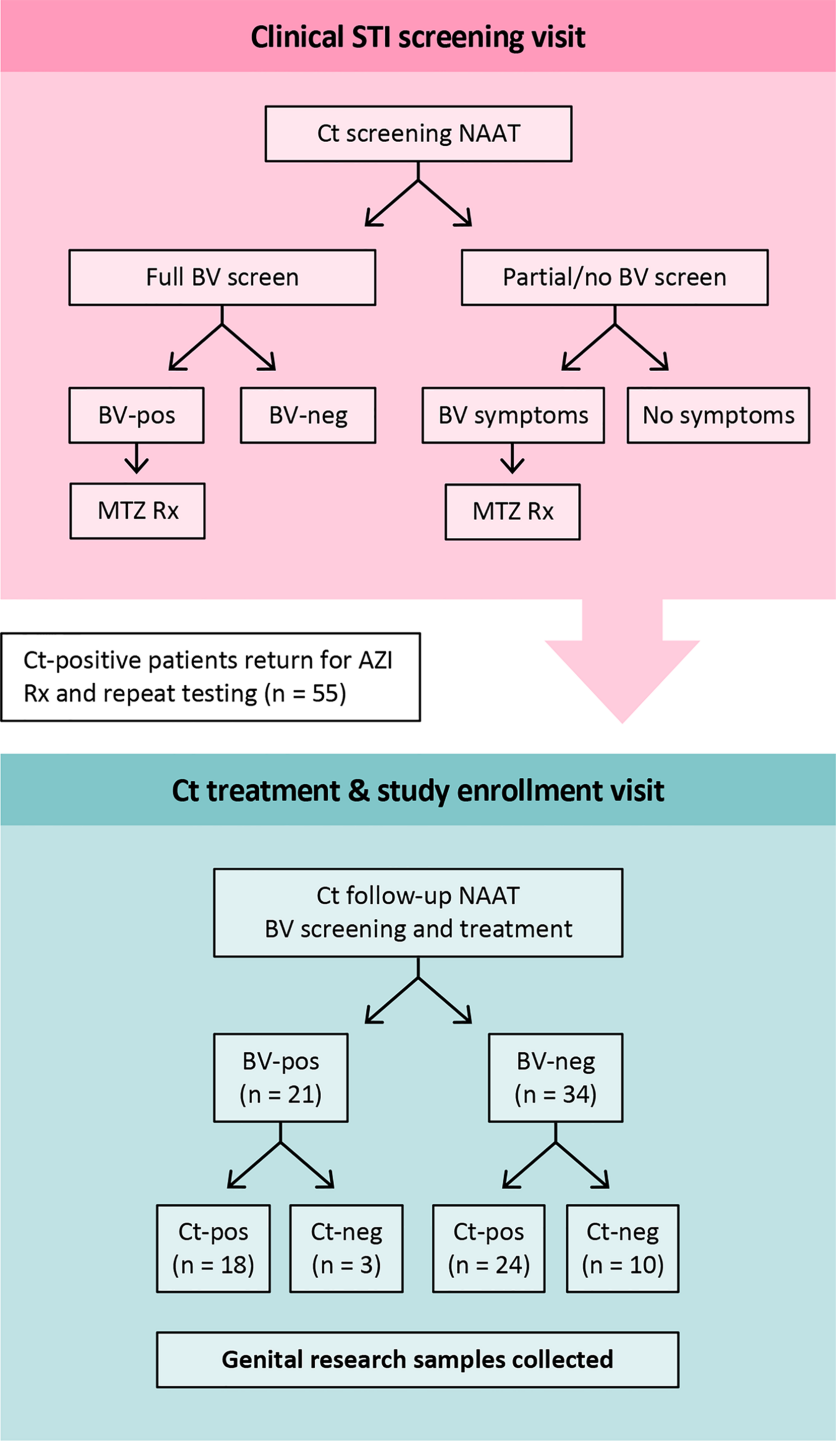
Study design. At the Ct screening visit, BV-positive was defined as an Amsel score of ≥3 of 4 by clinical assessment. All patients who were BV-Amsel positive after a full BV screen (n=20), or who were symptomatic for BV but with partial Amsel assessment (n=9), were prescribed metronidazole. All were compliant. For the Ct-treatment visit, when participants were enrolled, BV-positive was defined as a Nugent score of 7-10 by Gram stain. Abbreviations: MTZ, metronidazole; AZI, azithromycin.

Research samples from 55 women returning to the clinic for Ct treatment for a recently diagnosed Ct infection were analyzed. Twenty-seven were assessed for BV-Amsel at their Ct screening visit; 20 (74.1%) were positive (≥3 of 4 criteria), 19 were prescribed metronidazole or metronidazole gel treatment per CDC guidelines and 18 were adherent; 7 (25.9%) were BV-Amsel negative. The remaining 28 (50.1%) women were either not assessed or assessed by partial Amsel criteria for BV-Amsel at the Ct screening visit; in the latter group 9/28 (32.1%) were symptomatic for BV and therefore metronidazole treated for presumptive BV and 7 were adherent (**Figure 1**). The cohort was predominantly young (median age 24) and African American (76.4%) with half (50.9%) documenting a previous Ct infection (**Table 1**).

Women were classified as natural Ct clearers based on a negative Ct NAAT and undetectable cultivable Ct (IFU) at their Ct treatment visit. The natural Ct clearance rate was 13/55 (23.6%), and the time from the Ct screening to the Ct treatment visit was a median of 9 days (range 3-31 days). Women with a persisting Ct NAAT positive infection had an infectious Ct burden (median IFU 9,834 range 0 to 747,404) similar to our historic cohorts (Ficarra et al., 2008; Ibana et al., 2012). No significant difference was observed between Ct clearers and non-clearers with respect to number of days between screening and treatment/enrollment; age; black race; ≥1 partner in the last 30 days; prior known history of Ct infection; use of hormonal contraception; mucopurulent cervicitis or rates of *T. vaginalis*, yeast, *M. genitalium* or Herpes Simplex Virus (**Table 1**).

### Successful Metronidazole Treatment of BV May Permit More Women to Clear Ct Infection

Caldwell et al. hypothesized that a BV coinfection could allow Ct to escape natural eradication *in vivo* (Caldwell et al., 2003). We therefore determined if a BV diagnosis at the Ct treatment visit would predict Ct persistence. No significant difference was noted between Ct clearers and non-clearers when BV status at the Ct treatment visit was classified BV-Amsel (3-4) or BV-Nugent (7-10), (p=0.30 and p=0.33, respectively (**Table 1**)). We next determined if there were differences between Ct clearers and non-clearers when this was classified by a successful BV treatment response, using the following criteria: (1) Metronidazole treatment (500 mg orally twice/day for 7 days or metronidazole gel 0.75%, 5 g intravaginally once/day for 5 days) prescribed at the screening visit, (2) documented adherence to treatment, and (3) successfully resolution of BV by Nugent or Amsel criteria at the Ct treatment visit. However, BV treatment responders were no more common in Ct clearers than in non-clearers either by BV-Amsel or BV-Nugent (p>0.99 and p=0.36, respectively) (**Table 1**).

**Figure 2:**
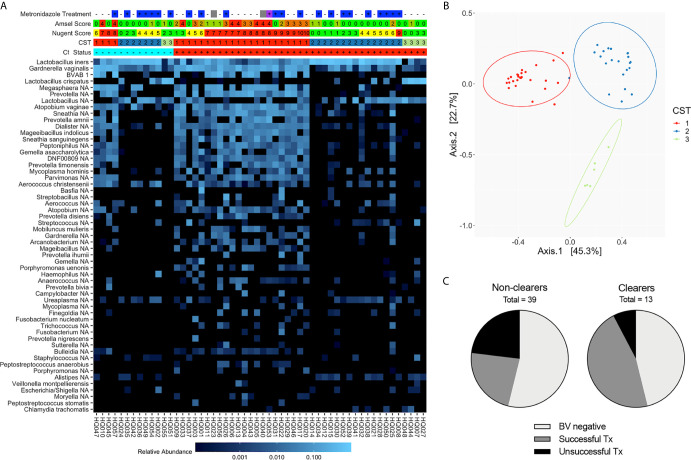
Composition and structure of the vaginal microbiota at the Ct treatment/research enrollment visit (n=54). **(A)** Heatmap of the most abundant bacterial taxa identified by 16S rRNA V4 sequencing of vaginal swabs from Ct clearing and non-clearing women. Women are categorized by BV status using Amsel (0-4), Nugent (green, 0-4; yellow, 4-6; red, 7-10), and CST (1-3). Metronidazole treatment at the Ct treatment visit is noted (–, untreated; +, treated; blank, NA; blue, metronidazole; purple, Metrogel; gray, treated for BV but unknown if this was metronidazole or metronidazole gel). Ct status is + and red for Ct positive and – and blue for Ct clearer. The scale bar demonstrates relative abundance of each species. Results indicate that an optimal *Lactobacillus*-dominated microbiome is not necessary to clear Ct. **(B)** Principal coordinates analysis demonstrating clustering into three distinct CSTs. **(C)** Pie chart depicting the portion of Ct clearers and non-clearers that were clinically BV negative at the screening visit or their BV resolution status following metronidazole treatment; those women that did not adhere to metronidazole treatment were omitted.

Since 16S rRNA gene sequencing provides a more comprehensive view of the vaginal microbiota composition than Amsel or Nugent, we next categorized Ct treatment visit samples by CST. Observed bacterial communities were then clustered into 3 distinct CSTs; CST3 was primarily composed of *L. crispatus* (11% of samples), CST2 was *L. iners* dominant (41% of samples) and CST1 lacked one clearly dominant species, though the majority were anaerobes known to be associated with BV (48% of samples) (**Figures 2A, B**). While a higher proportion of Ct clearers had a *Lactobacillus* spp.-dominant community (69% versus 46% of non-clearers) at their Ct treatment visit, not all were a CST2 or 3, and no significant difference was observed between *Lactobacillus* spp.-dominant and non-dominant CST groupings in clearers versus non-clearers. Within the Ct clearers, 6 out of 7 (85.7%) women successfully resolved BV with treatment in contrast to the non-clearer population in which 9/18 (50%) of those that were metronidazole treated did not resolve BV. In summary, while not required, a BV negative status, or successful metronidazole treatment of BV in the interim between screening and treatment for Ct, suggests this may aid more women to clear Ct infection (**Figures 2A, C**).

### BV Bacteria Are Found in the Endocervix of Ct Patients

**Figure 3:**
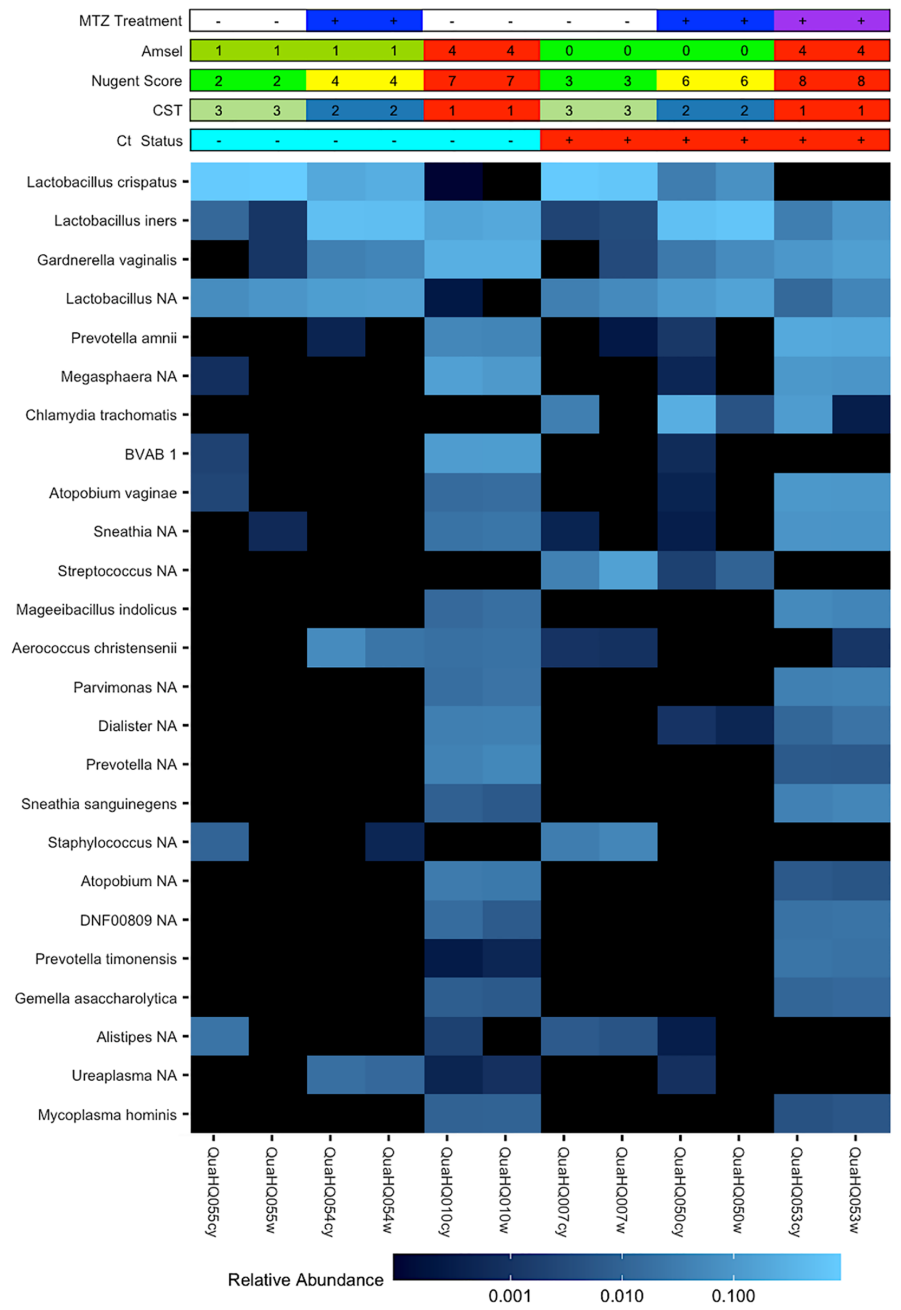
Paired cervical and vaginal microbiome samples. Heatmap of 25 most abundant bacterial taxa identified by 16S rRNA gene V4 sequencing of vaginal or cervical swabs from Ct clearing and non-clearing women. Matched samples are representative of the three CST groups and demonstrate the similarities in relative abundance of organisms between the vaginal and cervical environments within a woman. *Chlamydia trachomatis* is a dominant organism in the cervical samples of Ct non-clearers.

The circumvention of host immunity by BV bacteria or their products would require their accessibility to endocervical-located Ct. The availability of paired endocervical and vaginal-derived samples from a subset of this cohort enabled us to directly determine if BV bacteria could be found in the endocervix and if there were differences in relative bacterial abundances between the two sites given the distinct physiological differences. We observed similar relative abundances of bacteria between the cervix and vagina within the same woman, except for Ct which was a dominant organism of the endocervix of non-clearers as would be expected (**Figure 3**).

### IFNγ Is Present in the Genital Secretions of Clearers and Levels Are Not Significantly Different From Non-Clearers

**Figure 4:**
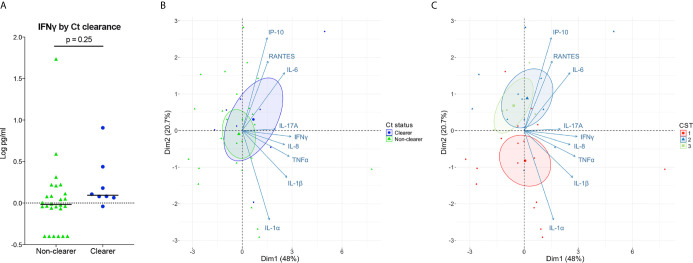
Associations of Ct clearance and CSTs with genital inflammation. **(A)** Log_10_-transformed IFNγ levels plotted by Ct clearance indicate clearers have slightly higher levels of IFNγ than non-clearers. **(B, C)** PCA performed over the log_10_ transformed cytokine values show a large overlap between Ct clearers and non-clearers in cytokines **(B)**. When colored by CST the PCA performed over the log transformed cytokine values shows a distinction between the *Lactobacillus* spp.-dominated CST2 and CST3 which associate more with IP-10, IL-6, and RANTES compared to the BV-like CST1 **(C)**.

*In vitro* models consistently demonstrate that sufficient and sustained IFNγ exposure can eradicate Ct in human cervical epithelial cells. Given this, we next determined whether clearing women had higher local levels of IFNγ illustrative of a more robust immune response, compared to women with persisting infection. Low levels of IFNγ in cervicovaginal lavages were observed in all women, irrespective of clearance status, and IFNγ levels were similar between clearers and non-clearers (**Figure 4A**; median, 1.2 *v* 1.0 pg/mL; p=0.25). In sum, women that clear Ct generally have lower levels of IFNγ than that required to clear Ct *in vitro*; therefore, IFNγ potentially could act in concert with additional factors in the local environment to clear Ct *in vivo*.

### Large Variations in Genital T Cell and Pro-Inflammatory Cytokines Associate With Microbiomes That Are Not Dominated by *Lactobacillus spp*

While IFNγ is considered a major cytokine involved in Ct clearance and protection, its levels are low in the lumen (Arno et al., 1990; Jordan et al., 2017), making biologically relevant comparisons difficult. Therefore, we expanded our cytokine panel to include IFNγ-induced proinflammatory cytokines and chemokines as a proxy for IFNγ activity, as well as other broadly Th1-classified cytokines and proinflammatory cytokines that have correlated with positive outcomes and/or protection. We performed principal component analysis (PCA) on the normalized cytokine concentrations to reduce the dimensionality of the dataset and to identify any cytokine signatures associated with Ct clearance and with vaginal microbiota composition. Our results showed a large overlap of inflammatory markers in clearers and non-clearers, with the clearers demonstrating only a slight shift toward IL-6 and IP-10 (**Figure 4B** **;** **Supplemental Table 1**). In contrast, women within CST1 were characterized by a robust signature, correlating with inflammatory markers IL-1α and IL-1β, while *Lactobacillus* spp.-dominated CSTs 2 and 3 correlated more with IL-6 and IP-10 (**Figure 4C** **;** **Supplemental Table 2**). Overall, our results suggest that the vaginal microbiota may be a stronger predictor of genital cytokine signatures, which may more accurately explain differences in Ct clearance in those with Ct/BV coinfections.

## Discussion

Ct employs multiple tactics to evade innate and adaptive immune mechanisms making it challenging for the host to establish complete, protective and long-lasting immunity. Identification and analysis of a population with robust immunity is one strategy to approach vaccine development in a targeted manner *via* identification of immune correlates of protection. In a series of studies from one US STI clinic, a surprising ~20% of women naturally cleared cervical Ct infection in the interim between Ct screening and antibiotic treatment and were significantly protected from incident infection (Geisler et al., 2008; Geisler et al., 2013). In our pilot study, undertaken in a New Orleans STI Clinic with comparable demographics, we documented a similar Ct clearance rate of 23.6%. We undertook analyses on genital samples collected at the treatment visit, ~9 days following initial screening of Ct, to identify any differences in the vaginal environment of clearers and non-clearers that may provide clues to mechanisms of Ct clearance.

Our cohort was characterized by a high prevalence of BV at the Ct screening visit, which was promptly treated when diagnosed. This enabled us to evaluate the potential impact of BV and metronidazole treatment on Ct clearance at the Ct treatment visit. Initial analyses explored differences in BV status between Ct clearers and non-clearers at their Ct treatment visit using Amsel and Nugent criteria, both of which are used clinically to define BV. As studies progressed, we focused our analyses on the most specific method used to determine the composition of the vaginal microbiota, 16S RNA sequencing, which categorizes samples based on specific bacterial abundances. No significant difference was observed in BV prevalence between Ct clearers and non-clearers at the Ct treatment visit, but we did observe that Ct clearers were more likely to have a *Lactobacillus*-dominant vaginal microbiota after metronidazole treatment compared to non-clearers (86% vs 50%). This suggests that metronidazole-induced changes in the microbiome could aid in host-mediated eradication of Ct, an observation that needs to be followed up in a larger cohort of women. Presently, nitromidazoles are the main class of drug approved for BV treatment (Workowski et al., 2010). However, it is acknowledged that these drugs are less than optimal in the treatment of chronic BV given their generally temporary effect and resultant shift to *L. iners* rather than *L. crispatus* dominance (Joag et al., 2019), an environment previously shown to increase susceptibility to Ct (Edwards et al., 2019). Moving forward, it will be important to carefully dissect the complex vaginal milieu created by BV bacteria, and their associated metabolites, in Ct infections to design more targeted treatments for BV, which could also ensure consistent, effective immunity to Ct (Aiyar et al., 2014; Sherchand and Aiyar, 2019).

*In vitro* and animal studies indicate that IFNγ is a central immune mediator for Ct eradication (Byrne et al., 1986a; Byrne et al., 1986b; Beatty et al., 1994a; Cain and Rank, 1995; Igietseme et al., 1998; Morrison and Caldwell, 2002; Brunham and Rey-Ladino, 2005). In human epithelial cells, IFNγ induces IDO-1, which at sufficient and sustained concentrations can promote Ct eradication through tryptophan starvation (Byrne et al., 1986a; Carlin et al., 1989; Beatty et al., 1994a; Beatty et al., 1994b; Belland et al., 2003). However, genital Ct isolates can salvage indole, long-hypothesized to be provided by BV-associated bacteria, to make tryptophan (Fehlner-Gardiner et al., 2002). In our cohort, the levels of IFNγ measured were significantly below those needed to clear Ct infections in *in vitro* models (Shemer and Sarov, 1985; Arno et al., 1990), suggesting additional factors may be at work *in vivo* in women. *In vitro* studies by Ziklo et al. have shown that Ct can induce IDO1 expression in the absence of IFNγ, further suggesting alternative regulatory pathways for IDO-1-induced tryptophan degradation (Ziklo et al., 2019). Additionally, Jordan et al. found decreased IFNγ concentrations in those that cleared Ct compared to those with a persistent infection, which could reflect the decreased production of IFNγ once Ct is cleared (Jordan et al., 2017). Although the interval between screening and treatment is similar in the two cohorts (9 days vs. 10 days, respectively), the exact day of clearance is unknown; therefore, conflicting IFNγ levels may reflect variation in times at which Ct antigen was cleared. This indicates the need to sample women prior to clearance to more definitively associate factors impeding or promoting clearance *in vivo*.

We expanded our cytokine study to include a panel of IFNγ-induced cytokines and chemokines, and proinflammatory cytokines classically used as markers of vaginal immunity and inflammation. We saw only small and non-significant differences in cytokine signatures between Ct clearers and non-clearers. In contrast, PCA plots were unique in women with CST1, driven by high levels of IL-1α and IL-1β and low levels of IP-10, a T cell chemokine previously reported to be inversely associated with BV and positively associated with *Lactobacillus* spp. including *L. iners* (Joag et al., 2019; Masson et al., 2019). We are intrigued by this finding as we previously reported that Ct abrogates endocervical epithelial cell secretion of IP-10 *in vitro* (Buckner et al., 2013), a finding recently confirmed by others (Antonia et al., 2019). It is possible that IP-10, particularly if modulated by effects of metronidazole treatment, may result in a rapid influx of T cells to aid in Ct clearance. In summary, our findings indicate that the vaginal microbiota and BV treatment can drive major changes in the local genital cytokine milieu that may modulate Ct clearance and/or serve as useful biomarkers. However, our findings also illustrate how the vaginal microbiota can lead to misinterpretation of the drivers of local inflammation in Ct infected women. A limitation of our study is the limited taxonomic resolution provided by 16S rRNA gene sequencing of the V4 hypervariable region and potential misclassification of certain bacterial taxa using the SILVA database. Another limitation of our, and all comparable previous studies, is that data is generated from samples taken post Ct clearance. This could lead to false assumptions, particularly regarding the range and depth of local genital host responses that may rapidly wane once chlamydial antigen is removed. Ideally, host, Ct and microbiome-associated analyses should also be taken on samples taken just prior to Ct eradication. As a pilot study, our cohort is also small, making statistical analyses challenging. These limitations are currently being addressed by a new larger, more extensive study in our clinic. This should allow a more comprehensive analysis of cytokines in the local environment and which may reveal a signature associated with Ct clearance.

Overall, this pilot study indicates that the microbiome and BV treatment have the potential to modulate the outcome of immunity to Ct (Aiyar et al., 2014; Ziklo et al., 2016a; Ziklo et al., 2016b; Li et al., 2020). Evaluation of the microbiome and local environment immediately prior to clearance as well as post-clearance should identify the sequence of events leading to the eradication of Ct *in vivo*. Analysis of bacterial metabolites, particularly indole and its derivatives, could more completely test the hypothesis and uncover a mechanism for the ability of Ct to harness an environmental milieu and evade immune clearance. It may also reveal how some women could clear Ct infection with an anaerobe-dominant vaginal microbiome. If specific factors can be identified, then targeted and more effective treatments can be designed and administered. This pilot study serves as preliminary data for forthcoming studies that focus on the optimal microenvironment, including the microbiome, immune milieu, and metabolome for effective Ct immunity.

## Data Availability Statement

The datasets presented in this study can be found in online repositories. The names of the repository/repositories and accession number(s) can be found below: https://www.ncbi.nlm.nih.gov/, PRJNA668201.

## Ethics Statement

The studies involving human participants were reviewed and approved by LSU Health Sciences Center Human Research Ethics Committee. The patients/participants provided their written informed consent to participate in this study.

## Author Contributions

AQ, DM, and CT conceived and designed the study. PM, CT, RL, CA, HA, and ML participated in data acquisition. PM, CT, CA, HA, KC, DM, LM, and AQ contributed to data analysis. PM, CT, and AQ prepared the report for publication. All authors contributed to the article and approved the submitted version.

## Funding

The study was funded by NIH grant AI118860.

## Conflict of Interest

The authors declare that the research was conducted in the absence of any commercial or financial relationships that could be construed as a potential conflict of interest.

## Supplementary Material

Supplementary Table 1 Vaginal cytokine concentrations by Ct status. Values indicate median (range) in pg/ml.

Supplementary Table 2 Vaginal cytokine concentrations by CST. Values indicate median (range) in pg/ml.
