# Supplementary material for: Differences in the Genital Microbiota in Women Who Naturally Clear Chlamydia trachomatis Infection Compared to Women Who Do Not Clear; A Pilot Study
Source: Front Cell Infect Microbiol. 2021 Apr 12;11:615770. doi: 10.3389/fcimb.2021.615770 (PMC8072278; doi:10.3389/fcimb.2021.615770)
Supplement: Supplementary Table 1 — Vaginal cytokine concentrations by Ct status. Values indicate median (range) in pg/ml. [file Table_1.pdf]

Cytokine	Clearers (n=8)	Persisting (n=26)	P-value
IFNγ	1.24 (0.91-8.09)	0.97 (0.4-54.53)	0.25
IL-17A	1.72 (0.35-62.66)	0.90 (0.35-43.24)	0.08
IL-1α	121.89 (28.38-1285.74)	121.46 (4.7-11643.96)	0.66
IL-1β	8.59 (1.32-182.72)	10.12 (0.4-2805.42)	0.96
IL-6	3.84 (1.61-120.35)	2.53 (0.45-112.42)	0.38
IL-8	1283.26 (139.95-6836.67)	516.98 (27.97-8754.11)	0.27
IP-10	111.46 (26.82-13658.07)	67.70 (9.06-4097.62)	0.37
MIP-1α	1.45 (1.45-46.27)	2.51 (1.45-126.49)	0.92
MIP-1β	1.5 (1.5-50.3)	1.5 (1.5-96.93)	0.84
RANTES	11.565 (1.7-135.17)	7.01 (0.6-405.25)	0.74
TNFα	1.025 (0.35-16.33)	0.83 (0.35-71.74)	0.50
