# Supplementary material for: Differences in the Genital Microbiota in Women Who Naturally Clear Chlamydia trachomatis Infection Compared to Women Who Do Not Clear; A Pilot Study
Source: Front Cell Infect Microbiol. 2021 Apr 12;11:615770. doi: 10.3389/fcimb.2021.615770 (PMC8072278; doi:10.3389/fcimb.2021.615770)
Supplement: Supplementary Table 2 — Vaginal cytokine concentrations by CST. Values indicate median (range) in pg/ml. [file Table_2.pdf]

Cytokine	BV-CST negative (n=17)	BV-CST positive (n=17)	P-value
IFNγ	1.2 (0.4-8.09)	0.95 (0.4-54.53)	0.94
IL-17A	0.93 (0.35-62.66)	1.15 (0.35-43.24)	0.69
IL-1α	71.06 (20.42-1728.35)	350.50 (4.7-11643.96)	0.00003
IL-1β	8.12 (0.4-120.81)	14.43 (1.70-2805.42)	0.009
IL-6	4.56 (0.91-120.35)	1.84 (0.45-112.42)	0.06
IL-8	564.51 (103.79-6836.67)	606.27 (27.97-8754.11)	0.67
IP-10	329.45 (26.82-13658.07)	51.92 (9.06-245.83)	0.0003
MIP-1α	1.45 (1.45-46.27)	1.45 (1.45-126.49)	0.52
MIP-1β	1.5 (1.5-50.3)	1.5 (1.5-96.93)	0.93
RANTES	7.56 (1.67-405.25)	7.67 (0.6-202.49)	0.39
TNFα	1 (0.35-16.33)	0.89 (0.35-71.74)	0.32
